# Cases of Venereal Inflammation of the Testis

**Published:** 1831-01-01

**Authors:** 


XXVI.

### VENEREAL INFLAMMATION OF THE TESTIS.

FROM the analysis of Sir A. Cooper's work, in the review department of the last and present Number, it will be seen that that experienced surgeon has described a venereal inflammation of the testicle. We had not room for several of the cases he brings forward in support of his opinions in that part, and we therefore supply the deficiency by inserting the particulars here.

*Case* 1. A. B. æt. 32, has the epididymis on the right side greatly enlarged and very much hardened—pain in the head and limbs aggravated to a distracting degree at night—painful node on the left tibia—pain and enlargement of the ulna. Four years and a half ago he had a chancre, for which he took a little mercury, and the disease disappeared. He continued well for some time, when a swelling began in his groin, which soon disappeared also. From that time he has had pains in his bones, relieved or not as he took or discarded medicine. The epididymis and testicle have been enlarged for twelve months, and though now not painful they were so at first.

*Case* 2. A gentleman who had been frequently subject to sore throats, which Sir Astley thought venereal, had his testis gradually enlarge without pain. Its shape was pyramidal, and not being able to distinguish it from hydrocele, our author put a lancet into the tunica vaginalis, when only two or three drops of blood were discharged. The oxymuriate, in tincture of bark, speedily reduced the enlargement of the testicle.

*Case* 3. A man applied to Sir Astley in Nov. 1827, with a testis diseased and hard as marble. Four years previously he had a venereal complaint, and, in a few weeks afterwards, the testis became enlarged, but was reduced in a month, under the use of mercury. In four months, the swelling in the testicle returned, and in two months it again disappeared by the same treatment. Two years ago it again swelled, and was again relieved. In the last Spring it became again swollen, and now, in the month of November, it is of large size. The mouth was for some time affected with mercury, but Sir Astley's notes do not contain the termination of the case.

*Case* 4. A. B. had a chancre three years ago, which was not succeeded by bubo. A year ago he had a hydrocele on the right side, which disappeared under the use of mercury and evaporating lotions. Seven or eight months ago he observed a swelling in the right testicle, which still continues; it is extremely solid, the epididymis is enlarged, the scrotum red, and there is pain in the loins and groin.

*Case* 5. C. T. has enlargement of both testes, without redness of the scrotum, and with very little pain. Sir

A. C. considered them venereal. He took large quantities of the decoctum sarsaparillæ compositum, with the oxymurias hydrargyri, and got perfectly well.

*Case* 6. A gentleman's servant had a chancre and bubo twelve months ago, since which time the left testis had become enlarged, very much hardened, and accompanied with hydrocele—mercury was given and he recovered.

Two more cases are related, but these we have noticed elsewhere. Whether it be thought that the testis is susceptible of true syphilitic enlargement, in connexion with secondary symptoms, or whether it be considered that the venereal virus merely gives occasion to chronic inflammation, it is satisfactory to know that mercury is the specific in both cases. In cachectic habits it should be combined with sarsaparilla.

